# Supplementary material for: Inhibitory Effect of Styrylpyrone Extract of Phellinus linteus on Hepatic Steatosis in HepG2 Cells
Source: Int J Mol Sci. 2023 Feb 12;24(4):3672. doi: 10.3390/ijms24043672 (PMC9959220; doi:10.3390/ijms24043672)
Supplement: Supplementary file 1 [file ijms-24-03672-s001.zip › ijms-2216746-supplementary.pdf]

Supplementary material

Inhibitory Effect of Styrylpyrone Extract of *Phellinus linteus* on Hepatic Steatosis in HepG2 Cells

Chun-Hung Chiu [1,2,†], Ming-Yao Chen [3,4,†], Jun-Jie Lieu [1], Chin-Chu Chen [5], Chun-Chao Chang [3,6,7,*], Charng-Cherng Chyau [1,*] and Robert Y. Peng [1,8]

1 Research Institute of Biotechnology, Hungkuang University, Shalu District, Taichung City 43302, Taiwan
2 Department of Program in Animal Healthcare, Hungkuang University, Shalu District, Taichung City 43302, Taiwan
3 Division of Gastroenterology and Hepatology, Department of Internal Medicine, School of Medicine, College of Medicine, Taipei Medical University, Taipei 11031, Taiwan
4 Division of Gastroenterology and Hepatology, Department of Internal Medicine, Taipei Medical University- Shuang-Ho Hospital, New Taipei City 235041, Taiwan
5 Grape King Biotechnology Center, Longtan Dist., Taoyuan 325002, Taiwan
6 Division of Gastroenterology and Hepatology, Department of Internal Medicine, Taipei Medical University Hospital, Taipei 11031, Taiwan
7 TMU Research Center for Digestive Medicine, Taipei Medical University, Taipei 110, Taiwan
8 Graduate Institute of Clinical Medicine, College of Medicine, Taipei Medical University, Taipei 110301, Taiwan
* Correspondence: chunchao@tmu.edu.tw (C.-C. C.); ccchyau@hk.edu.tw (C.-C.C.); Tel.: +886-4-26318652 (Charng-Cherng Chyau); Fax: +886-4-26525386 (Charng-Cherng Chyau)
† These authors contributed equally to this work.

Table S1. List of primer pairs used for StepOnePlus™ Real-Time PCR System.

Gene		Sequence (5' to 3')
β-actin	F	CCCAGCACAATGAAGATCAAGATCAT
	R	ATCTGCTGGAAGGTGGACAGCGA
Sirt-1	F	ACAGGTTGCGGGAATCCAAAGG
	R	CCTAGGACATCGAGGAACTACCTG
NF-κB	F	AACAGCAGATGGCCCATACC
	R	AACCTTTGCTGGTCCCACAT
PGC1-α	F	AGACACCGCACGCACCGAAAT
	R	AGCTGTCATACCTGGGCCGACG
AMPK	F	TTTGCGTGTACGAAGGAAGAAT
	R	CTCTGTGGAGTAGCAGTCCCT
